# Engineering Nonvolatile Polarization in 2D α-In_2_Se_3_/α-Ga_2_Se_3_ Ferroelectric Junctions

**DOI:** 10.3390/nano15030163

**Published:** 2025-01-22

**Authors:** Peipei Li, Delin Kong, Jin Yang, Shuyu Cui, Qi Chen, Yue Liu, Ziheng He, Feng Liu, Yingying Xu, Huiyun Wei, Xinhe Zheng, Mingzeng Peng

**Affiliations:** Beijing Key Laboratory for Magneto-Photoelectrical Composite and Interface Science, School of Mathematics and Physics, University of Science and Technology Beijing, No. 30, Xueyuan Road, Beijing 100083, China; m202210750@xs.ustb.edu.cn (P.L.); d202410764@xs.ustb.edu.cn (D.K.); d202210420@xs.ustb.edu.cn (J.Y.); m202310753@xs.ustb.edu.cn (S.C.); u202242156@xs.ustb.edu.cn (Q.C.); m202310770@xs.ustb.edu.cn (Y.L.); u202242160@xs.ustb.edu.cn (Z.H.); u202242159@xs.ustb.edu.cn (F.L.); xuyingying@ustb.edu.cn (Y.X.); huiyunwei@ustb.edu.cn (H.W.)

**Keywords:** group-III selenides, 2D ferroelectrics, polarization engineering, band alignments, strain modulation

## Abstract

The advent of two-dimensional (2D) ferroelectrics offers a new paradigm for device miniaturization and multifunctionality. Recently, 2D α-In_2_Se_3_ and related III–VI compound ferroelectrics manifest room-temperature ferroelectricity and exhibit reversible spontaneous polarization even at the monolayer limit. Here, we employ first-principles calculations to investigate group-III selenide van der Waals (vdW) heterojunctions built up by 2D α-In_2_Se_3_ and α-Ga_2_Se_3_ ferroelectric (FE) semiconductors, including structural stability, electrostatic potential, interfacial charge transfer, and electronic band structures. When the FE polarization directions of α-In_2_Se_3_ and α-Ga_2_Se_3_ are parallel, both the α-In_2_Se_3_/α-Ga_2_Se_3_ P↑↑ (UU) and α-In_2_Se_3_/α-Ga_2_Se_3_ P↓↓ (NN) configurations possess strong built-in electric fields and hence induce electron–hole separation, resulting in carrier depletion at the α-In_2_Se_3_/α-Ga_2_Se_3_ heterointerfaces. Conversely, when they are antiparallel, the α-In_2_Se_3_/α-Ga_2_Se_3_ P↓↑ (NU) and α-In_2_Se_3_/α-Ga_2_Se_3_ P↑↓ (UN) configurations demonstrate the switchable electron and hole accumulation at the 2D ferroelectric interfaces, respectively. The nonvolatile characteristic of ferroelectric polarization presents an innovative approach to achieving tunable n-type and p-type conductive channels for ferroelectric field-effect transistors (FeFETs). In addition, in-plane biaxial strain modulation has successfully modulated the band alignments of the α-In_2_Se_3_/α-Ga_2_Se_3_ ferroelectric heterostructures, inducing a type III–II–III transition in UU and NN, and a type I–II–I transition in UN and NU, respectively. Our findings highlight the great potential of 2D group-III selenides and ferroelectric vdW heterostructures to harness nonvolatile spontaneous polarization for next-generation electronics, nonvolatile optoelectronic memories, sensors, and neuromorphic computing.

## 1. Introduction

With the advancement in semiconductor technology, high-density storage, and computing integrated applications, ferroelectric materials and devices are continuously pursuing miniaturization. However, as dimensions shrink, traditional ferroelectrics, including BiFeO_3_ [1], BaTiO_3_ [2], PbTiO_3_ [3]_,_ etc., encounter the challenge of size effects. As a result, the exploration of ultrathin ferroelectric materials remains the key issue in this field. Two-dimensional ferroelectrics possess inherently stable layered structures, characterized by strong intralayer bonding, weak interlayer vdW interactions, and high electron mobility due to their unique quantum size effects. These attributes offer novel opportunities for advancing the exploration of 2D ferroelectricity and its extensive applications at the atomic scale. The symmetry breaking of 2D ferroelectrics has been achieved through various strategies, such as stacking vdW heterostructures, applying strains, and engineering Janus structures [4,5]. Recently, 2D ferroelectrics such as 1*T*-MoS_2_ [6], α-In_2_Se_3_ [7], CuInP_2_S_6_ [4], and MX (M = Ge, Sn; X = S, Se, Te) [8] have gained tremendous interest due to their unique features in contrast to 3D counterparts. The breakthrough of 2D ferroelectricity has driven emerging applications in 2D ferroelectric field-effect transistors [9,10], ferroelectric tunnel junctions [11], and high-photosensitivity and rapid-response photodetectors [12]. As we are entering into the post-Moore era, the discovery of 2D ferroelectrics with desired performance characteristics becomes crucial for advancing the frontiers of electronic device technology.

As part of the 2D ferroelectric family, 2D III–VI layered semiconductors have garnered extensive attention. Specifically, they exhibit five distinct structural phases (α, β, γ, δ, κ). Among these, α, β, and γ are common phases [13,14], and δ and κ are uncommon phases [15,16]. The γ phase is identified as a three-dimensional material with a hexagonal defect wurtzite crystal structure. In contrast, the other phases including α, β, δ, and κ exhibit a layered structure [17]. For instance, α-In_2_Se_3_ is the most stable layered structure with spontaneous ferroelectric polarization at room temperature [7]. Two-dimensional α-In_2_Se_3_ has been experimentally and theoretically confirmed to possess a robust out-of-plane ferroelectric and piezoelectricity polarization [18,19,20]. The monolayer α-In_2_Se_3_ is alternately composed of Se and In atomic layers through covalent bonding to form a quintuple-layer structure of Se–In–Se–In–Se [21]. The unequal interlayer distances between the middle Se layer and its adjacent In layers lead to the breaking of symmetry. Several research groups have subsequently confirmed the spontaneous in-plane and out-of-plane ferroelectricity of ultrathin α-In_2_Se_3_ at room temperature [19,22] and fabricated high-performance rigid and flexible optoelectronic detectors [23]. Similar to other polar materials, symmetry breaking in α-In_2_Se_3_ leads to the misalignment of positive and negative charge centers, producing a dipole moment and spontaneous polarization. The out-of-plane intrinsic polarization is reversible, permitting the manipulation of ferroelectric polarization in both upward and downward directions by laterally shifting the central Se layer. When integrated with other polar semiconductors, 2D α-In_2_Se_3_ can induce charge redistribution and further manipulate electronic energy properties. It was found that the interfacial dipole interaction plays a crucial role in the external electric field modulation, with a direct correlation to the charge distribution at α-In_2_Se_3_/GaN polar heterointerfaces [24]. The asymmetric metal/α-In_2_Se_3_/Si crossbar ferroelectric semiconductor junctions have been demonstrated to enhance the modulation of the effective Schottky barrier height by the ferroelectric polarization [25]. The inherent nonvolatile ferroelectric switching and semiconducting characteristics of α-In_2_Se_3_ show versatile fields including nonvolatile memory, neuromorphic computing, optoelectronics, and thermoelectric applications [15,26]. As another member of 2D III–VI layered semiconductors, α-Ga_2_Se_3_ has been demonstrated to possess excellent structural stability, which is obtained by replacing In of α-In_2_Se_3_ with Ga. The first-principles calculations have indicated that α-Ga_2_Se_3_ similar to α-In_2_Se_3_ is a semiconductor with Young’s modulus of less than 100 N m^−1^ in a deformation range of up to about 30% [27]. The switched polarization was observed in 2D α-Ga_2_Se_3_ nanoflakes of approximately 4 nm with a high switching temperature of up to 450 K [28]. Such polarization switching could arise from the displacement of Ga vacancy between neighboring asymmetrical sites by applying an electric field [28]. In the graphene/α-Ga_2_Se_3_ vdW heterojunctions, the presence of spontaneous polarization reveals intriguing interfacial characteristics by controlling contact types and external disturbances for the multifunctional design of high-performance Schottky devices [29]. The ferroelectric nature of α-Ga_2_Se_3_ has opened avenues for its use in nonvolatile memory devices and non-linear optical applications. Other studies have also reported that α-Ga_2_Se_3_ exhibits significant piezoelectric performance, showing broad prospects in ultrathin piezoelectric sensors and nanogenerators [30]. Although the α-In_2_Se_3_ and α-Ga_2_Se_3_ layered materials have been extensively studied, the limitation of 2D ferroelectric multilayers and complex stacking heterostructures impedes a comprehensive understanding of their intricate polarization interactions, polarity modulation mechanisms, and synergistic effects. Therefore, the exploration of 2D group-III selenide ferroelectrics and polar vdW heterostructures holds profound significance, extending 2D ferroelectrics and semiconducting optoelectronics by offering novel functionalities and characteristics that surpass those of traditional 3D ferroelectrics.

In this study, we have proposed 2D α-In_2_Se_3_/α-Ga_2_Se_3_ ferroelectric vdW heterostructures and investigated the ferroelectric polarization modulation by first-principles calculations. Considering the reversible ferroelectric polarization of α-In_2_Se_3_ and α-Ga_2_Se_3_, the positive and negative c-axes are defined as polarity up (P↑) and polarity down (P↓), respectively. In terms of polarity manipulation, there are switchable ferroelectric polarization configurations including α-In_2_Se_3_/α-Ga_2_Se_3_ P↑↑ (UU), α-In_2_Se_3_/α-Ga_2_Se_3_ P↑↓ (UN), α-In_2_Se_3_/α-Ga_2_Se_3_ P↓↑ (NU), and α-In_2_Se_3_/α-Ga_2_Se_3_ P↓↓ (NN). Our calculations demonstrate that the UN and NU configurations exhibit a pronounced accumulation of holes and electrons at the ferroelectric heterointerfaces of α-In_2_Se_3_/α-Ga_2_Se_3_, facilitating ferroelectric modulation of conductivity types between the p-channel and n-channel within FeFETs. In addition, the electronic band structures of the α-In_2_Se_3_/α-Ga_2_Se_3_ polar heterostructures have been effectively manipulated by employing in-plane biaxial strain modulation. From compressive to tensile strains, their band alignments undergo a type III–II–III transition in UU and NN configurations, and a type I–II–I transition in UN and NU configurations. Strain engineering provides a versatile approach for controllable energy band transformation, facilitating the charge separation, transfer, recombination, and redistribution processes in 2D α-In_2_Se_3_/α-Ga_2_Se_3_ ferroelectric heterostructures. Therefore, 2D group-III selenides and ferroelectric vdW heterostructures provide atomic-level platforms that integrate electronic, optical, and memory functionalities for nonvolatile multifunctional applications.

## 2. Calculation Methods

**Figure 1:**
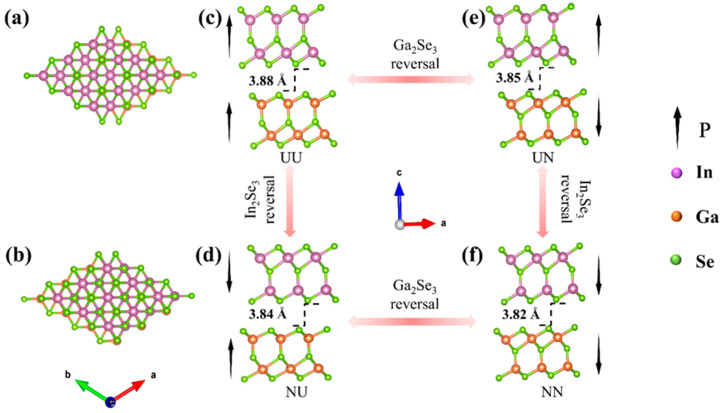
Two-dimensional α-In_2_Se_3_/α-Ga_2_Se_3_ ferroelectric heterostructures: top views of (**a**) UU and UN, (**b**) NU and NN, and side views of (**c**) UU, (**d**) NU, (**e**) UN, and (**f**) NN. The purple, orange, and green represent In, Ga, and Se atoms, respectively. The black arrow represents the direction of polarization.

First-principles calculations were carried out using the Vienna ab initio Simulation Package (VASP) simulation method based on density functional theory (DFT) [31,32]. To calculate the electron exchange–correlation interactions, we employed the meta-generalized gradient approximation (meta-GGA) and the Strongly Constrained and Appropriately Normed (SCAN) functional to calculate the geometry optimizations and electronic band structures [33,34]. Compared to the local density approximation (LDA) and the Perdew, Burke, and Ernzerhof (PBE) method, meta-GGA SCAN has been tested in diversely bonded systems, where it was shown to be sophisticated enough to model a wide range of physical structures without being fitted to any bonded system [35]. It can describe an intermediate range of dispersion via the kinetic energy density and can be proven to deliver sufficiently accurate ground-state properties for diversely bonded systems [36,37]. The projector-augmented wave (PAW) method was used to describe the ion–electron interactions [38,39]. For the calculations, we constructed α-In_2_Se_3_/α-Ga_2_Se_3_ (hexagonal, space group *R*3m) unit cells with different polarity configurations, as depicted in Figure 1. Their 2D ferroelectric polarization modulation has been investigated on the structural stability, electrostatic potential, interfacial charge transfer, and electronic band structures by both the ferroelectric polarization reversals of α-In_2_Se_3_ and α-Ga_2_Se_3_, which serve as a foundation for the exploration of 2D ferroelectric/optoelectronic multifunctional applications. We utilized a plane-wave basis expansion with a cutoff energy of $E_{cut}$ = 500 eV and employed a 12 × 12 × 1 Monkhorst–Pack K point grid in the Brillouin Zone. And, Grimme’s DFT-D3 vdW corrections were incorporated based on the semiempirical GGA-type theory [40]. A vacuum layer of 15 Å along the c direction was adopted to ensure decoupling between periodically repeated systems. For the relaxation of the unit cells at zero strain, all atoms were fully relaxed with a convergence accuracy of 1 × 10^−8^ eV. The interaction force between atoms was found to be less than |0.02| eV/Å. When considering the strain modulation on the α-In_2_Se_3_/α-Ga_2_Se_3_ ferroelectric heterojunctions, the in-plane biaxial strains (ɛ) in the a and b directions are defined as follows: ɛ = 100% × (a_1_ − a_0_)/a_0_, where a_1_ and a_0_ represent the lattice parameters with and without applied biaxial strains, respectively.

## 3. Results and Discussion

### 3.1. Polarity Configuration and Structural Stability

To investigate the electronic properties of 2D α-In_2_Se_3_/α-Ga_2_Se_3_ ferroelectric heterostructures, the fully-optimized lattice constants are a = b = 4.03 Å for α-In_2_Se_3_ and a = b = 3.84 Å for α-Ga_2_Se_3_, respectively, which are in good agreement with previous reports [9,41]. The small lattice mismatch between α-In_2_Se_3_ and α-Ga_2_Se_3_ is 4.8%, demonstrating good feasibility to construct their vdW heterointerfaces with low strains and few defects. In addition, different stacking orders between α-In_2_Se_3_/α-Ga_2_Se_3_ need to be considered, as shown in Figure 1, including the In–Se, In–Ga, and Se–Se alignments. The structural stability of each stacking order has been examined by calculating the binding energy ($E_{b}$) of 2D α-In_2_Se_3_/α-Ga_2_Se_3_ ferroelectric heterostructure systems as follows:

$$E_{b}=E_{In2Se3/Ga2Se3}-E_{In2Se3}-E_{Ga2Se3},$$

where $E_{In2Se3/Ga2Se3}$, $E_{In2Se3}$, and $E_{Ga2Se3}$ are the total energies of α-In_2_Se_3_/α-Ga_2_Se_3_ heterostructures, α-In_2_Se_3_, and α-Ga_2_Se_3_, respectively. As shown in Figure 1a,b, it is found that the In–Ga alignment has the smaller binding energy in α-In_2_Se_3_ (P↑)/α-Ga_2_Se_3_ configurations, while the In–Se alignment has the smaller binding energy in α-In_2_Se_3_ (P↓)/α-Ga_2_Se_3_ configurations. The stacking order has a direct change from In–Ga to In–Se due to switching the α-In_2_Se_3_ polarization from upward to downward. Based upon the above results, the four most stable α-In_2_Se_3_/α-Ga_2_Se_3_ ferroelectric heterostructures have been achieved in Figure 1c–f by stacking α-In_2_Se_3_ (P↑) on α-Ga_2_Se_3_ (P↑) (UU), α-In_2_Se_3_ (P↑) on α-Ga_2_Se_3_ (P↓) (UN), α-In_2_Se_3_ (P↓) on α-Ga_2_Se_3_ (P↑) (NU), and α-In_2_Se_3_ (P↓) on α-Ga_2_Se_3_ (P↓) (NN), respectively. It should be noted that the NN configuration has the shortest vdW interlayer distance. Therefore, it demonstrates that the stacking order in combination with ferroelectric polarization plays an important influence in 2D interfacial interactions, such as polarization coupling, electrostatic interaction, charge transfer, photoelectric conversion, etc. [42,43].

### 3.2. Band Structures and Density of States

**Figure 2:**
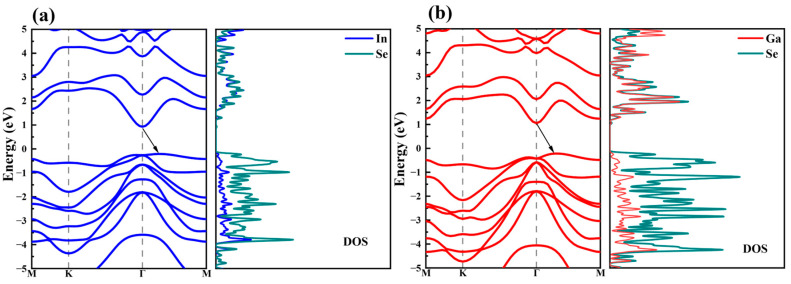
Projected band structures and DOS of (**a**) α-In_2_Se_3_ and (**b**) α-Ga_2_Se_3_. The Fermi level is set as 0.

As shown in Figure 2a,b, the projected electronic band structures and density of states (DOS) of monolayer α-In_2_Se_3_ and α-Ga_2_Se_3_ have been firstly calculated by using the SCAN function. Both of them have similar band structures with the conduction band minimum (CBM) located at the Γ point and the valence band maximum (VBM) positioned between the Γ and M points. The bandgaps of α-In_2_Se_3_ and α-Ga_2_Se_3_ are 1.14 eV and 1.27 eV, respectively, and both exhibit an indirect bandgap. According to the DOS results, the electronic states at both the CBM and VBM are predominately attributed to the Se atoms, in good consistence with the previous results [23].

**Figure 3:**
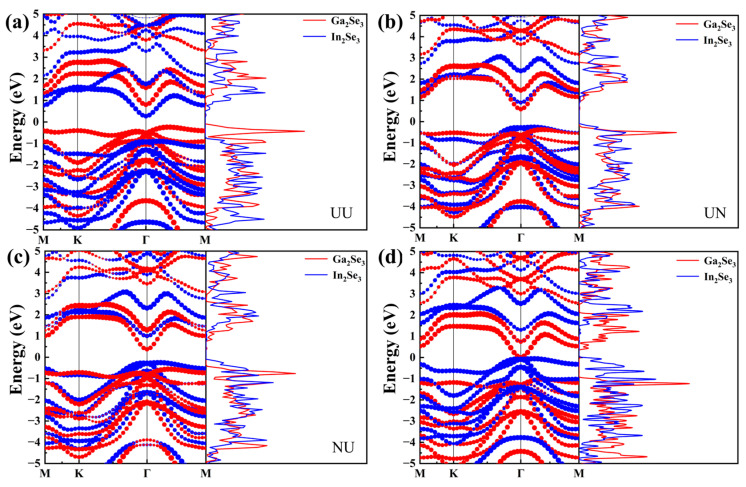
Projected band structures and DOS of the α-In_2_Se_3_/α-Ga_2_Se_3_ heterojunctions in (**a**) UU, (**b**) UN, (**c**) NU, and (**d**) NN. The blue and red marks represent the contributions of the α-In_2_Se_3_ and α-Ga_2_Se_3_ layers on projected band structures and projected density of states, respectively. The Fermi level is set as 0.

When two different materials (namely, A and B) contact each other, the band alignment types are based on the classification of semiconductor heterostructures. They are categorized into three types: type I (Straddling gap), type II (staggered gap), and type III (broken gap). In type I, the conduction band minimum (CBM) of band B is higher in energy than the CBM of band A, and the valence band maximum (VBM) of band B is lower in energy than the VBM of band A. This results in a straddling gap where the energy levels of bands A and B overlap. In type II, both the CBM and VBM of band B are higher in energy than the CBM and VBM of band A, respectively. A staggered gap is formed where their energy levels do not overlap. In type III, the conduction band minimum of band A is lower in energy than the VBM of band B, resulting in a broken gap. To further clarify the ferroelectric polarization interactions, Figure 3 shows the projected band structures and DOS of 2D ferroelectric α-In_2_Se_3_/α-Ga_2_Se_3_ heterostructures. The red and blue lines correspond to the projected weights of electrons for α-Ga_2_Se_3_ and α-In_2_Se_3_, respectively. By switching the ferroelectric polarization state of α-Ga_2_Se_3_ from P↑ to P↓, the electronic energy bands of α-Ga_2_Se_3_ and α-In_2_Se_3_ experience pronounced downward and upward shifts in opposite trends, respectively. They are both observed during the configuration transitions from UU to UN in Figure 3a,b and from NU to NN in Figure 3c,d. Consistently, upon reversing the ferroelectric polarization state of α-In_2_Se_3_ from P↑ to P↓, the electronic energy bands of α-Ga_2_Se_3_ and α-In_2_Se_3_ undergo similar bandshifts from UU to NU or from UN to NN, as depicted in Figure 3a and c or Figure 3b and d, respectively. The UU, UN, NU, and NN configurations have indirect bandgaps of 0.51 eV, 0.85 eV, 0.63 eV, and 0.08 eV, respectively. Although all of them exhibit type-II alignments, the CBM and VBM originate from different ferroelectric layers of α-In_2_Se_3_ and α-Ga_2_Se_3_. Specifically, the heterojunction bands come from the CBM of α-In_2_Se_3_ and the VBM of α-Ga_2_Se_3_ in UU and from the VBM of α-In_2_Se_3_ and the CBM of α-Ga_2_Se_3_ in NN. As a result, the tunable type-II alignments can not only drive the spontaneous separation of free electrons and holes but also determine the charge transfer directions by reversing the 2D ferroelectric polarization field. It is noted that the CBM of α-Ga_2_Se_3_ and VBM of α-In_2_Se_3_ in NN approach the Fermi level, resulting in the largest bandshifts, as shown in Figure 3d. This highlights the significant role of polarity manipulation in controlling the electronic energy bands of 2D ferroelectric heterojunctions.

### 3.3. Electrostatic Potential and Charge Transfer

**Figure 4:**
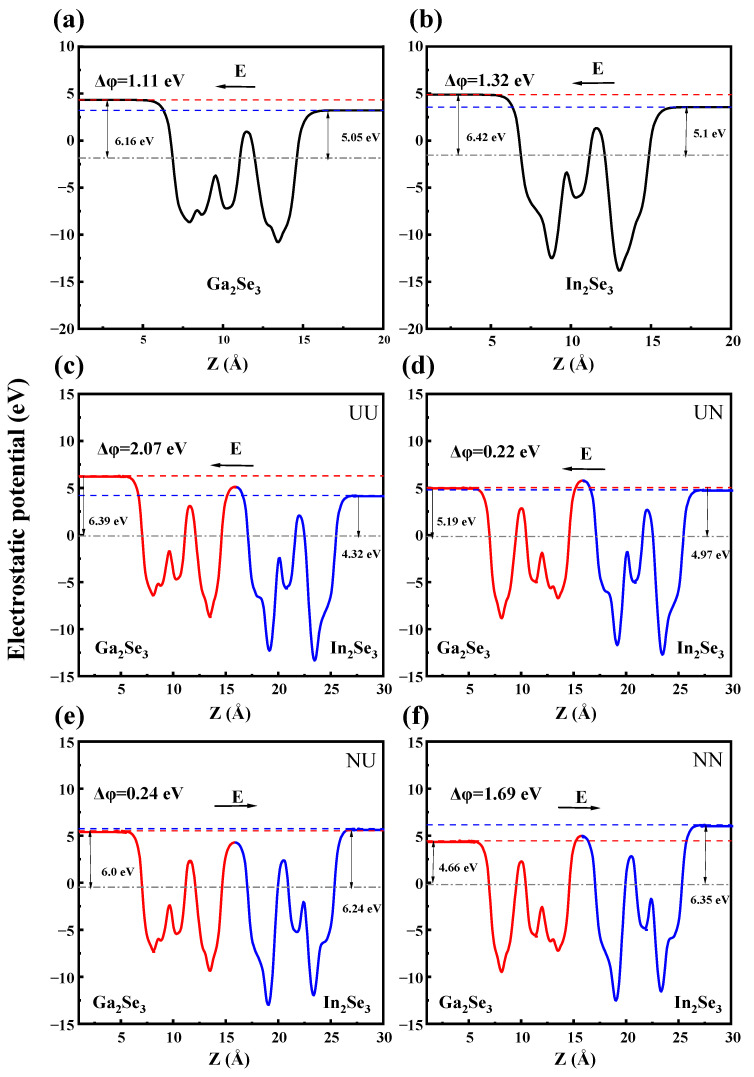
Planar average electrostatic potentials of (**a**) α-In_2_Se_3_, (**b**) α-Ga_2_Se_3_, (**c**) NN, (**d**) UN, (**e**) NU, and (**f**) NN configurations heterostructures along the z-direction, respectively. In (**c**–**f**), the blue and red parts of the electrostatic potential curves correspond to α-In_2_Se_3_ and α-Ga_2_Se_3_, respectively. The arrows indicate the directions of the polarization-induced electric fields. The vacuum levels and Fermi levels are marked with blue, red, and gray dashed lines, respectively.

**Figure 5:**
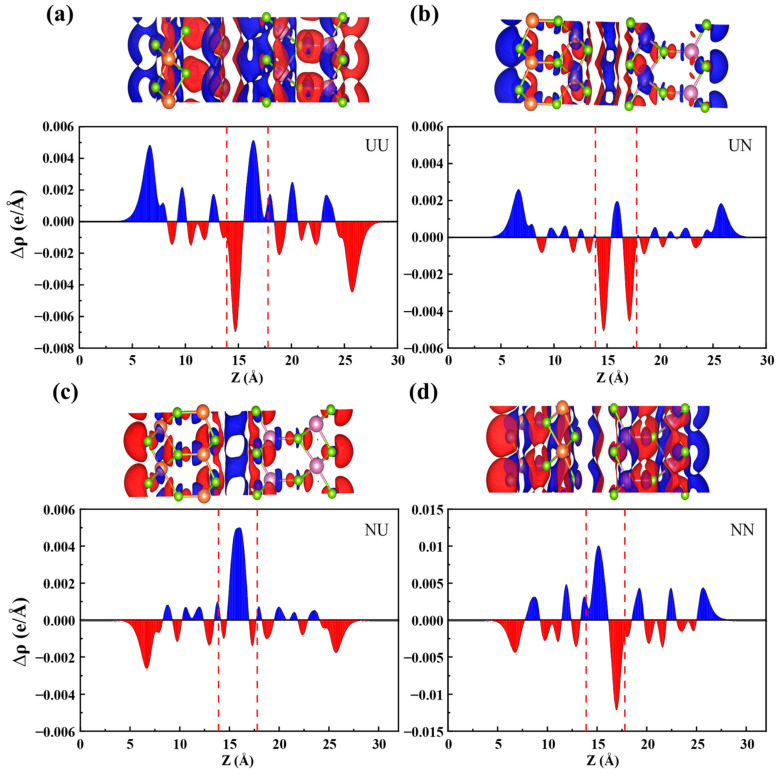
Plane-averaged charge density differences of the α-In_2_Se_3_/α-Ga_2_Se_3_ heterostructures in (**a**) UU, (**b**) UN, (**c**) NU, and (**d**) NN configurations along the z-direction, respectively. The red dashed lines represent the α-In_2_Se_3_/α-Ga_2_Se_3_ interface boundaries.

The electrostatic potential and charge transfer distribution are intricately linked to the ferroelectric polarization interactions at the 2D α-In_2_Se_3_/α-Ga_2_Se_3_ heterointerfaces, which in turn significantly impact their electronic energy bands and electrical transport behaviors. Figure 4 calculated the electrostatic potential distribution along the z-axis for α-In_2_Se_3_, α-Ga_2_Se_3_, and α-In_2_Se_3_/α-Ga_2_Se_3_ ferroelectrics in four stacking configurations, respectively. As an indicator of ferroelectric polarization strength, the electrostatic potential differences (ΔΦ) are 1.32 eV and 1.11 eV for α-In_2_Se_3_ and α-Ga_2_Se_3,_ as shown in Figure 4a and b, respectively. So, α-In_2_Se_3_ has a more robust ferroelectric polarization than α-Ga_2_Se_3_, in consistency with the different electronegativity between In and Ga. After contacting each other, the α-In_2_Se_3_/α-Ga_2_Se_3_ heterostructures in Figure 4c–f obtain the electrostatic potential differences of 2.07 eV (UU), 0.22 eV (UN), 0.24 eV (NU), and 1.69 eV (NN), respectively. In the UU and UN configurations, the electrostatic potential of α-Ga_2_Se_3_ P↑ or P↓ is higher than α-In_2_Se_3_, indicating that the polarization-induced electric field is directed from α-In_2_Se_3_ to α-Ga_2_Se_3_, as shown in Figure 4c,d. As shown in Figure 4e,f, when switching the ferroelectric polarization state of the α-In_2_Se_3_ layer from P↑ to P↓ in the NU and NN configurations, the electrostatic potential of α-Ga_2_Se_3_ P↑ or P↓ becomes lower than α-In_2_Se_3_, thereby reversing the direction of the polarization-induced electric field. When α-In_2_Se_3_ and α-Ga_2_Se_3_ are polarized in the same directions, the electrostatic potential differences of the UU and NN configurations are higher than that of each single material but are lower than the sum of both. In contrast, when polarized in opposite directions, the electrostatic potential differences of the UN and NU configurations are greatly reduced. In addition, the UN and NU ferroelectric heterointerfaces display markedly weaker polarization-induced fields compared to their UU and NN counterparts. It demonstrates that there exists a certain charge transfer, which partially offsets the electrostatic potential difference. Under the manipulation of ferroelectric polarization, the net charge transfer is also substantial to the electrostatic potential difference at ferroelectric heterointerfaces, thereby mitigating the total ferroelectric polarization strength. Therefore, it is noted that the opposite UN and NU polarization configurations may manifest strikingly distinct characteristics in ferroelectric interlayer interactions, charge accumulation or separation, and carrier transport behaviors compared to the identical UU and NN configurations. Based on Figure 5a,d, it can be observed that the accumulation and dissipation of charges at the interface are approximately equal for both UU and NN configurations. This suggests that the redistribution of charges primarily occurs at the interface.

The phenomenon of spontaneous polarization arises in both α-In_2_Se_3_ and α-Ga_2_Se_3_ as a direct consequence of central symmetry breaking. It is attributed to the spatial displacement of positive and negative charges, leading to a non-uniform charge distribution within these materials. Upon the formation of a heterojunction, the charge redistribution occurs across the entire α-In_2_Se_3_/α-Ga_2_Se_3_ ferroelectric heterointerfaces, leading to the formation of an interfacial dipole moment. The presence of the interfacial dipole moment induces a built-in polarization field at the 2D ferroelectric interface, which in turn affects the energy band alignments and the motion behaviors of electrons and holes. Specifically, to analyze the interfacial charge transfer after α-In_2_Se_3_ and α-Ga_2_Se_3_ contact together, Figure 5 presents the charge density differences ($∆\rho \left(z\right)$) of 2D α-In_2_Se_3_/α-Ga_2_Se_3_ heterostructures in different stacking configurations. It is defined as

$$∆\rho \left(z\right)=\int \rho_{hetero}\left(x,y,z\right)dxdy-\int \rho_{In2Se3}\left(x,y,z\right)dxdy-\int \rho_{Ga2Se3}\left(x,y,z\right)dxdy,$$

where $\rho_{hetero}(x,y,z)$, $\rho_{In2Se3}(x,y,z)$, and $\rho_{Ga2Se3}(x,y,z)$ are the charge densities of α-In_2_Se_3_/α-Ga_2_Se_3_ heterostructures, α-In_2_Se_3_, and α-Ga_2_Se_3_ at the (x, y, z) position, respectively. The blue and red colors represent the charge accumulation and depletion, respectively. The ferroelectric polarization is directed from the negatively charged center to the positively charged center. In the UU configuration of Figure 5a, the negative polarization surface of α-In_2_Se_3_ is in contact with the positive polarization surface of α-Ga_2_Se_3_ at its 2D ferroelectric interface. And, the interfacial dipole interaction prompts the electrons and holes to separate apart from each other and accumulate at both ends of the UU heterojunction. It facilitates the rapid separation of photogenerated electron–hole pairs, causing the electrons in the α-Ga_2_Se_3_ layer to be repelled to the bottom surface and accumulate there. At the same time, the positive charge center of the α-Ga_2_Se_3_ layer is adjacent to the α-In_2_Se_3_ layer, causing the electrons of the α-In_2_Se_3_ layer to be attracted and accumulate near the interface. In contrast, due to the positive polarization surface of α-In_2_Se_3_ being adjacent to the negative polarization surface of α-Ga_2_Se_3_, the internal built-in polarization field drives the electrons and holes in the opposite direction, as shown in the NN configuration of Figure 5d, which are finally accumulated on the top and bottom surfaces of α-In_2_Se_3_ and α-Ga_2_Se_3_, respectively. On the other hand, both the UN and NU configurations demonstrate quite weak built-in polarization fields at 2D α-In_2_Se_3_/α-Ga_2_Se_3_ heterointerfaces. As depicted in Figure 5b, there exist the negatively polarized charges of both α-In_2_Se_3_ (P↑) and α-Ga_2_Se_3_ (P↓) at the interface of the UN configuration, which results in a hole potential well to capture the massive holes and accumulate there. According to Figure 4d, the electrostatic potential barrier also causes the electrons to be repelled from the interface of the UN configuration. Conversely, the interface of the NU configuration exhibits the positively polarized charges of both α-In_2_Se_3_ (P↓) and α-Ga_2_Se_3_ (P↑). As shown in Figure 5c, it is capable of capturing and accumulating a substantial amount of electrons at the interface. There presents an electron potential well as observed in Figure 4e. As a result, the switchable accumulation of electrons and holes has been successfully achieved at 2D α-In_2_Se_3_/α-Ga_2_Se_3_ heterointerfaces by manipulating nonvolatile ferroelectricity. This method provides valuable insights into the conversion of channel conductivity types from electrons to holes for 2D FeFETs.

**Figure 6:**
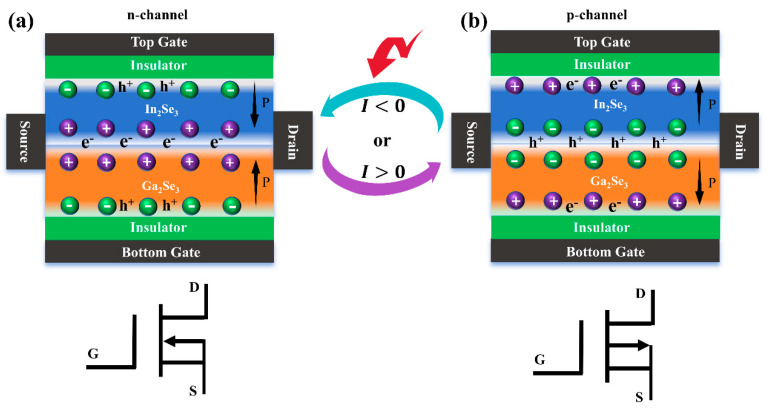
(**a**) The n-channel and (**b**) p-channel FeFET devices based on NU and UN configurations, respectively. The red arrow represents that the FeFET channel current flows in opposite directions. The “P” represents the ferroelectric polarization directions controlled by the top and bottom gates.

As is well known, FeFETs offer low power consumption during writing/reading operations with high rapidity and high scalability that is compatible with CMOS technology. Note that the ferroelectric polarization has been experimentally demonstrated to be reversible by an electric field [18,19]. By utilizing the reversible out-of-plane ferroelectric polarization, 2D α-In_2_Se_3_/α-Ga_2_Se_3_ FeFETs have been proposed in Figure 6. By leveraging the charge transfer properties of the NU and UN configurations, a ferroelectric reconfigurable FeFET device model has been developed, where the top gate and bottom gates are used to independently control the polarization states of α-In_2_Se_3_ and α-Ga_2_Se_3_, and the source and drain terminals are positioned on both sides of the α-In_2_Se_3_/α-Ga_2_Se_3_ heterointerfaces. When the polarization electric field is applied, the polarization state of the ferroelectric layer exhibits ferroelectric hysteresis, which remains unchanged even after removing the electric field. For the NU configuration in Figure 6a, the channel carriers are electrons, indicating an n-channel FeFET. At $V_{gs}=0$, the FeFET is conductive due to the presence of electrons in the channel. When increasing the negative bias voltage ${\;V}_{gs}<0$, the n-channel starts to narrow, and ultimately enters the cut-off state once approaching the threshold voltage. Conversely, by reversing both the top and down gates, the ferroelectric polarization of 2D α-In_2_Se_3_/α-Ga_2_Se_3_ FeFETs is changed to the UN configuration. As shown in Figure 6b, the p-channel FeFET is in a conductive state due to the presence of holes in the channel at $V_{gs}=0$. When further increasing the positive bias voltage ${\;V}_{gs}>0$, the p-channel starts to narrow, gradually approaches the threshold voltage, and eventually enters the cut-off state. By switching the polarization states from NU to UN, it is worth mentioning that the FeFET channel current flows in opposite directions. As a result, the nonvolatile nature of ferroelectric polarization enables a stable transition between the n-channel and p-channel of 2D α-In_2_Se_3_/α-Ga_2_Se_3_ FeFETs, which helps to achieve fully complementary logic components. The ferroelectric modulation strategy provides significant guidance and opportunities for advanced data storage and processing technologies in nonvolatile electronic logic and memory applications.

### 3.4. Strain Modulation on α-In_2_Se_3_/α-Ga_2_Se_3_ Polar Heterostructures

**Figure 7:**
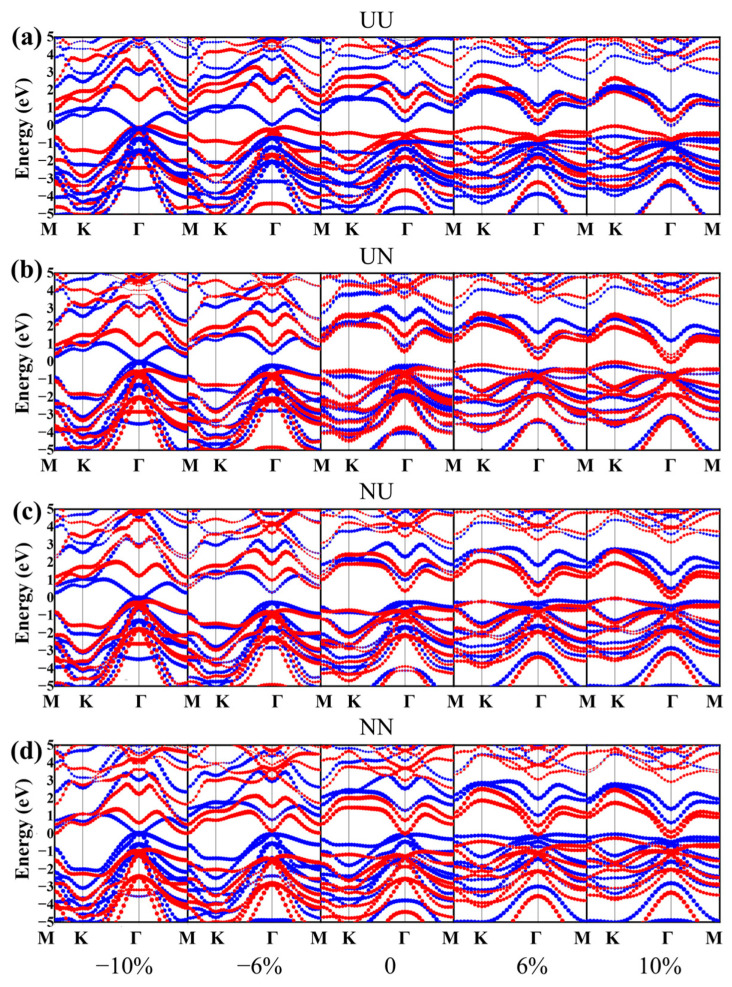
Projected band structures of (**a**) UU, (**b**) UN, (**c**) NU, and (**d**) NN heterostructures under biaxial strains of −10%, −6%, 0%, 6%, and 10%, in which the blue and red colors indicate the contributions of α-In_2_Se_3_ and α-Ga_2_Se_3_, respectively.

Owing to the exceptional mechanical flexibility and intrinsic piezoelectric characteristics of 2D ferroelectrics, strain engineering has emerged as a crucial technique for modulating their electronic band structures. In this study, we explored the modulation of the electronic characteristics of α-In_2_Se_3_/α-Ga_2_Se_3_ polar heterostructures by applying in-plane biaxial strains. Figure 7 shows the projected energy bands of α-In_2_Se_3_/α-Ga_2_Se_3_ polar heterostructures in UU, UN, NU, and NN configurations from −10% (compressive) to +10% (tensile), respectively. Because of belonging to the same III–VI family, it is noted that α-In_2_Se_3_ and α-Ga_2_Se_3_ have similar trends of electronic band structures on in-plane biaxial strains. As tensile strains increase, the CBMs of α-In_2_Se_3_ and α-Ga_2_Se_3_ decrease obviously while their VBMs gradually increase. It leads to decreasing bandgaps of both α-In_2_Se_3_ and α-Ga_2_Se_3_. In addition, the valence bands distinctly shift upward at the K point when applying tensile strains, leading to the VBM transition from the Γ point to the K point. On the other side, the bandgaps of α-In_2_Se_3_ and α-Ga_2_Se_3_ become narrower under high compressive strains. The substantial structural transformation results in changing the atomic interactions and making a distinct shift in charge distribution and band structures.

**Figure 8:**
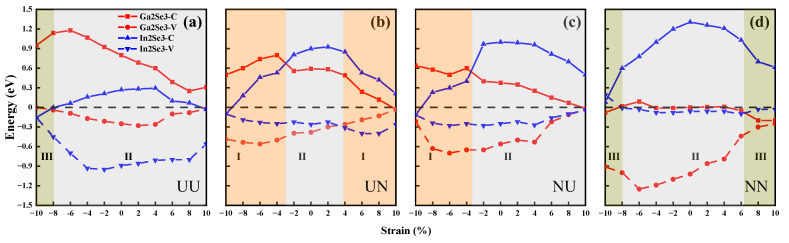
CBM and VBM values of α-In_2_Se_3_/α-Ga_2_Se_3_ heterostructures in (**a**) UU, (**b**) UN, (**c**) NU, and (**d**) NN configurations as a function of the in-plane biaxial strain, respectively. The red (blue) solid lines and dashed lines represent the CBMs and VBMs of α-Ga_2_Se_3_ (α-In_2_Se_3_), respectively. The orange, gray, and light green regions denote type-I, type-II, and type-III band structures, respectively.

Furthermore, the band alignments of α-In_2_Se_3_/α-Ga_2_Se_3_ polar heterostructures are highly sensitive to the ferroelectric polarization configurations. When changing the configurations from UU to UN, NU, and NN in Figure 7, the electronic energy bands of α-Ga_2_Se_3_ and α-In_2_Se_3_ shift downward and upward significantly in opposite trends, respectively. As the compressive or tensile strain increases in Figure 8a,d, the CBM of α-In_2_Se_3_ becomes lower than the VBM of α-Ga_2_Se_3_ in the UU configuration, whereas the CBM of α-Ga_2_Se_3_ becomes lower than the VBM of α-In_2_Se_3_ in the NN configuration. As shown in Figure 8a,d, both the UU and NN configurations experience a band alignment transition spanning from type III to type II and back to type III. Conversely, in UN and NU configurations, the CBM and VBM of α-In_2_Se_3_ are positioned intermediately between those of α-Ga_2_Se_3_ under compressive strain, and the CBM and VBM of α-Ga_2_Se_3_ are situated between those of α-In_2_Se_3_ under tensile strain. Figure 8b,c demonstrate that both the UN and NU configurations undergo a band alignment transition covering type I→II→I. Consequently, strain can profoundly influence the shape of electronic band structures, modulate the bandgap values and their direct or indirect types, and tune the band alignment relationships in the α-In_2_Se_3_/α-Ga_2_Se_3_ ferroelectric heterostructures. The controllable transformation of energy bands facilitates the charge separation, transfer, recombination, and redistribution processes in the α-In_2_Se_3_/α-Ga_2_Se_3_ ferroelectric heterostructures. As a result, 2D III–VI ferroelectric junctions integrate electronic, optical, and memory capabilities for nonvolatile multifunctional applications.

## 4. Conclusions

In summary, the structural stability, electrostatic potential, interfacial charge transfer, and electronic band structures of 2D α-In_2_Se_3_/α-Ga_2_Se_3_ ferroelectric heterostructures have been thoroughly investigated by using first-principles calculations. The results indicate that α-In_2_Se_3_ has a stronger ferroelectric polarization compared to α-Ga_2_Se_3_. The polarization reversal of α-In_2_Se_3_ facilitates the nonvolatile manipulation of the built-in electric field within α-In_2_Se_3_/α-Ga_2_Se_3_ ferroelectric heterointerfaces, allowing for the reversible field directions. The pronounced built-in electric field in the UU and NN prompts the electrons and holes to separate apart from each other, leading to free carrier depletion. Conversely, the switchable accumulation of electrons and holes has been successfully achieved at the 2D ferroelectric interfaces of NU and UN. The nonvolatile nature of ferroelectric polarization enables a novel approach for modulating the electrical conductivity transition between the n-channel and p-channel in 2D α-In_2_Se_3_/α-Ga_2_Se_3_ FeFETs. Moreover, the band alignments of the α-In_2_Se_3_/α-Ga_2_Se_3_ ferroelectric heterostructures have been modulated by in-plane biaxial strains, achieving a type III–II–III transition in UU and NN, and a type I–II–I transition in UN and NU, respectively. Our findings demonstrate that 2D ferroelectric vdW heterostructures open promising avenues to utilize nonvolatile spontaneous polarization for the development of advanced data logic, optoelectronic memory, and computing techniques.

## Data Availability

The data that support the findings of this study are available from the corresponding author upon reasonable request.
